# A Laboratory Evaluation of the New Automated Pollen Sensor Beenose: Pollen Discrimination Using Machine Learning Techniques

**DOI:** 10.3390/s23062964

**Published:** 2023-03-09

**Authors:** Houssam El Azari, Jean-Baptiste Renard, Johann Lauthier, Thierry Dudok de Wit

**Affiliations:** 1 LPC2E-CNRS, 3A Avenue de la Recherche Scientifique, CEDEX 2, 45071 Orléans, France; 2 LIFY-AIR, Le LAB’O, 1 Avenue du Champ de Mars, 45100 Orléans, France; 3 ISSI, Hallerstrasse 6, 3012 Bern, Switzerland

**Keywords:** pollen monitoring, real time, optical sensor, machine learning

## Abstract

The monitoring of airborne pollen has received much attention over the last decade, as the prevalence of pollen-induced allergies is constantly increasing. Today, the most common technique to identify airborne pollen species and to monitor their concentrations is based on manual analysis. Here, we present a new, low-cost, real-time optical pollen sensor, called Beenose, that automatically counts and identifies pollen grains by performing measurements at multiple scattering angles. We describe the data pre-processing steps and discuss the various statistical and machine learning methods that have been implemented to distinguish different pollen species. The analysis is based on a set of 12 pollen species, several of which were selected for their allergic potency. Our results show that Beenose can provide a consistent clustering of the pollen species based on their size properties, and that pollen particles can be separated from non-pollen ones. More importantly, 9 out of 12 pollen species were correctly identified with a prediction score exceeding 78%. Classification errors occur for species with similar optical behaviour, suggesting that other parameters should be considered to provide even more robust pollen identification.

## 1. Introduction

Anemophilous plants liberate large quantities of pollens to ensure their reproduction, exceeding 500 billion grains per individual for some tree species [1,2]. This pollen dispersal in ambient air is a major cause of seasonal allergies worldwide and the role of pollen grains in triggering conjunctivitis, allergic rhinitis, or asthma is well established [3,4,5,6]. The number of people affected by pollen-induced allergies is significant, totalling approximately 400 million people in the world suffering from allergic rhinitis, and 300 million from asthma [7]. The consequences are deleterious health effects and a deterioration in quality of life of the patients [8,9], which results in substantial direct and indirect costs related, inter alia, to medication and patient care expenditures, absenteeism, and presenteeism [7,10,11,12,13]. At the same time, there is a growing body of evidence suggesting that the prevalence of these allergies will increase dramatically in the future with a worsening of symptoms, mainly owing to higher levels of atmospheric pollution and climate change [14,15,16,17,18]. The increase in CO_2_ concentrations together with the global rise in temperatures have been reported to enhance pollen production and allergenicity, to facilitate the spread of invasive plant species with a high allergenic potency (e.g., Ambrosia) and to alter plant development leading to earlier, longer, and more intense pollen seasons [17,19,20,21]. In this context, pollen monitoring is an issue of great importance.

To date, the most common technique to characterize airborne pollens and quantify them is based on volumetric samplers using either the Hirst design [22] or the Rotorod technology [23,24]. The measurement principle involves collecting pollen grains deposited on an adhesive surface, in general after a sampling period of one week, then identifying and counting them under a microscope to obtain the past week’s daily average pollen concentrations per specie. Such manual instruments have been used in at least 749 monitoring stations out of 879 across the world [25] and their wide use has progressively led to the development of standardized pollen monitoring methodologies as described in [26].

Notwithstanding their conventional nature, their wide use, and their ability to provide a big-picture view of the pollinic content of ambient air, the methodological limitations of these instruments raise questions about their effectiveness from a health and allergy management point of view. The task of identifying and counting pollen grains by eye via microscope is tedious and time demanding, which, when added to the sampling period of one week, generates a prejudicial time delay in disseminating the pollinic information [26]. Moreover, only 10% of the pollen grains deposited on the adhesive surface are usually analysed, generating high uncertainties in reported low concentrations [27]. Operating this type of instrument can also be subject to high overheads as highly skilled technicians are mobilized to carry out the analysis process manually, which restricts the possibility to deploy a dense network of monitoring stations essential to correctly cover a specific area. For instance, there are only 85 active pollen stations covering countries as large as USA or France [25] and monitored cities are rarely if ever equipped with more than one volumetric sampler. As a result, spatial heterogeneity of pollen concentrations related to local sources of emissions—within one city—and reported by several studies [28,29,30] is not accounted for in the pollinic information provided to allergy sufferers. To address the above-mentioned issues at least in part, developing new automated solutions has become of great interest.

With recent advances in computer science and sensor technologies, several automatic pollen sensors have been developed over the past few years and are starting to be deployed either in routine monitoring or as part of validation campaigns. Some of these devices mimic the Hirst concept and fully automate the analysis process using image recognition techniques. In [31,32], the authors introduced the BAA500 system which identifies and counts the pollen grains deposited on a glass slide using a convolutional neural network. The algorithm is trained on a large library of microscopic images at multiple focal positions and is reported to identify 40 pollen species with a multiclass accuracy over 90%.

Other automatic devices are based on air-flow cytometry, with in most cases, a combination of machine learning algorithms such as convolutional neural networks and technologies as diverse as laser induced fluorescence, digital holography, or elastic light scattering [33,34,35,36]. Among these, one can cite the SwisensPoleno monitors [34] in which the pollen classification task is carried out using a deep convolutional neural network with a VGG16 architecture. The experimental results show that the device is able to recognize six pollen species out of eight with an accuracy above 90%.

Regardless of the technology used and despite their promising abilities in identifying specific pollen species and providing associated concentrations in real-time, these new automatic instruments are still too expensive and/or too cumbersome to be deployed in dense networks, which is an obstacle for more local and localized pollen information.

In our previous study [37], we showed that pollens exhibit specific intensity scattering curves allowing their detection among other types of particles and even to distinguish between their various families. In this paper we introduce Beenose, a new small and relatively low-cost pollen sensor that has been developed based on our previous results. Beenose aims at recognizing different pollen taxa and delivering their concentrations in real time using their multi-angular light scattering patterns. More specifically, we ran laboratory measurements in which we inserted different aerosols into the instrument. These include pollen samples of interest, droplets, and carbonaceous and mineral particles. By processing the instrument response, we obtained size distributions and optical signatures (hereafter called speciation indices) for each sample. 

The aim of the current paper is twofold. First, we assess the sensor’s ability in particle sizing by exploring the size distributions. Second, we examine the possibility of distinguishing between pollen and non-pollen particles and discriminating various pollen taxa using optical signatures. Such a laboratory work represents a prerequisite to deploying Beenose in an operational context by investigating the feasibility of laser optics-based pollen identification as an alternative to the conventional manual method.

## 2. Materials and Methods

### 2.1. Instrument Description

**Figure 1:**
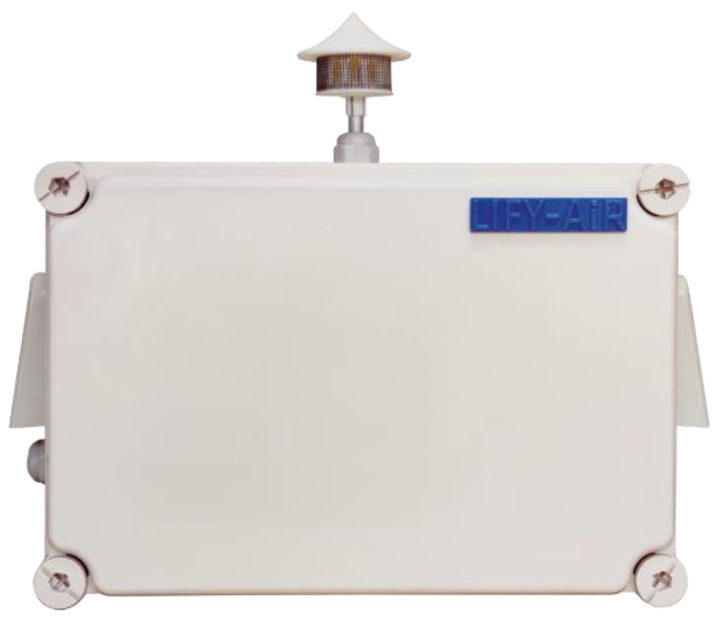

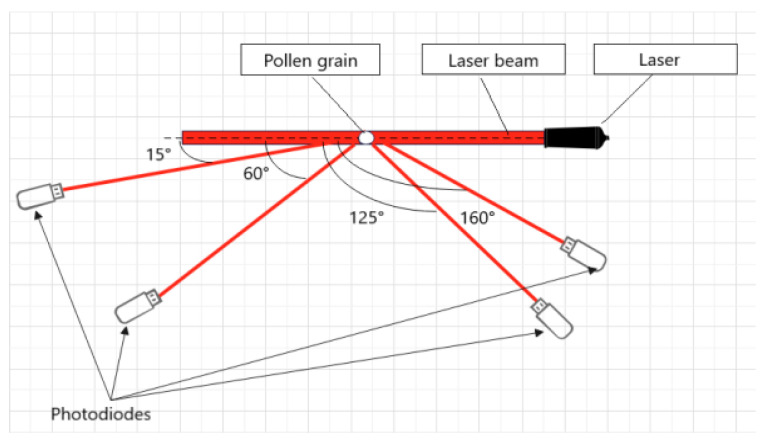
The Beenose sensor, with a picture of the instrument (**top panel**) and principle of measurement (**lower panel**).

Beenose is an optical pollen sensor developed by the Lify-Air company in collaboration with the French National Center for Scientific Research—CNRS—and manufactured by Lify-Air. The instrument is a 30 × 20 × 12 cm^3^ airflow cytometer (see Figure 1) that uses the principle of light scattering to identify and count pollens and other types of aerosols. Air is sampled via a metal profiled inlet and particles are drawn up to the optical chamber using a pump with a flow rate of 10 L/min. The particles cross a 650 nm laser beam and the light that is scattered by each of them is recorded by 4 photodiodes at scattering angles respectively of 15°, 60°, 125°, and 160° (hereafter named respectively, Channel 1 to Channel 4) to the laser axis as shown in Figure 1. Each channel produces light intensity distributions by counting and classifying particles in 19 predefined bins (each corresponding to a range of light intensities). Due to a low scattering angle of 15°, the scattered light recorded by the first channel is almost independent of the refractive index of irregular particles [38]. Thus, the first channel provides access to size distributions by counting and classifying pollen grains and other aerosols in 19 size bins between 5 µm and 100 µm. These size distributions are obtained thanks to a power law relationship between the intensity of the scattered light and the optical diameter of the particles. The 3 other channels (also named “speciation channels”) are on the contrary sensitive to the imaginary part of the refractive index and therefore more sensitive to particle parameters such as shape, surface properties, and light absorbance. Thus, the light intensity distributions provided by these speciation channels are used in comparison with that provided by the first channel to produce the optical signature.

### 2.2. Instrument Calibration

Prior to our experimentation, we calibrated the Beenose sensor to establish the relationship between the scattered flux and the electrical response (in mV) for calibrated irregular particles. We used slightly irregular beads and silicon carbide grains that are usually employed for polishing mirrors. Grains smaller than 20 μm were released in the air while those between 20 and 100 μm in size were directly injected in the instrument. Mineral particles of various origins and different sizes were also selected by a system of calibrated sieves and injected directly into the instrument to complete the calibration curves and ensure all the channels have the necessary sensitivity to detect these particles. Intercomparison sessions with the LOAC aerosol counter [39] were carried out to check the accuracy of Beenose in terms of particle counting.

### 2.3. Pollen Samples and Laboratory Measurements

To evaluate the ability of Beenose to discriminate different types of pollens for allergy monitoring purposes, we explored 12 pollen species that were selected for their allergic potency and/or for being part of the species that are routinely monitored by existing monitoring networks. The pollen taxa were supplied by the Stallergenes Greer Company (in the US) in a dry state with exhaustive information such as pollen size, collection method, and origin of the plant. A summary of the pollen samples used in this study is given in Table 1.

For each pollen sample we performed measurement sessions in a dedicated room; the apparatus was systematically cleaned before and after each measurement session by using an air compressor. Pollen grains were released for suspension above a fan placed 1 m from the device inlet. We inspected pollen counts in real time to avoid a saturation of the instrument and to ensure enough particles were still in suspension; otherwise, the injection process was repeated. The number of samples and the total number of grains that were measured for each pollen species are reported in Table 1.

For each pollen sample, we recorded light intensity distributions provided every 10 s by the four channels during a period ranging from 30 to 50 min. This resulted in 12 raw datasets with a number of rows varying from 179 to 299 (the number of samples), and 76 columns (4 channels × 19 light intensity bins). Note that in addition to the pollen samples, we also recorded the signal of carbonaceous particles, mineral particles, and droplets to verify whether they had distinct optical responses.

### 2.4. Data Processing

**Figure 2:**
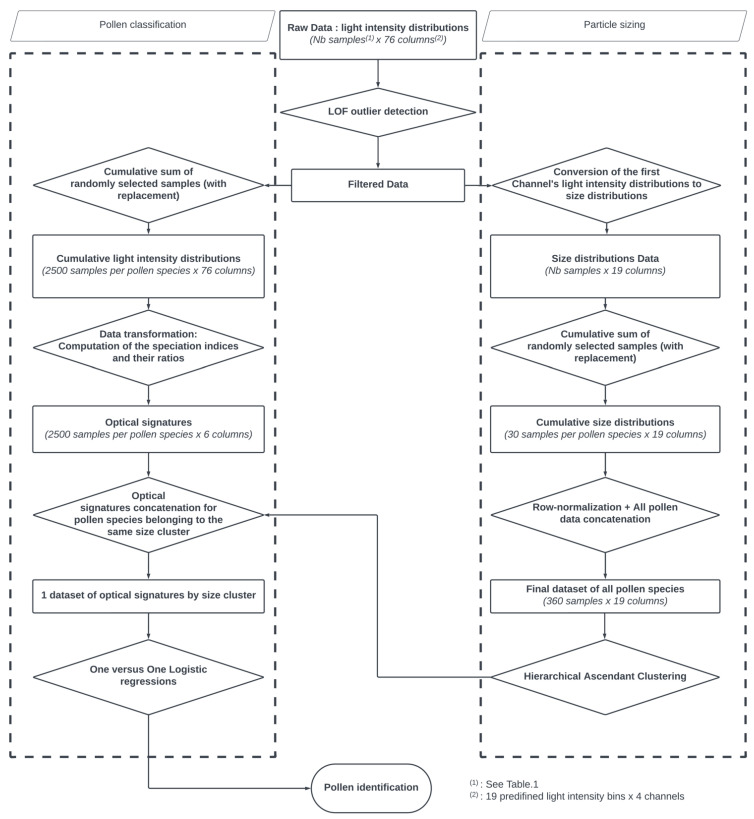
An outline of the proposed methodology.

The flowchart of the proposed methodology is illustrated in Figure 2.

#### 2.4.1. Particle Sizing

Our first objective was to evaluate the consistency of particle sizing by clustering the size distributions derived from the light intensity distributions of the first channel (see Section 2.1) and verifying whether the clusters matched the diameters of the pollen species, as provided by the supplier. Before we could do so, we had to pre-process the data in two steps.

First, after visually inspecting the counts recorded by the first channel, we found that our data were occasionally contaminated by outliers that could be ascribed to ambient air particles, pollen agglomerates, debris, and dirt. The latter three are probably due to the storage conditions of the pollen samples and their dry state. Such contaminated measurements could potentially distort both the size information provided by the sensor and the optical signatures. For this purpose, we implemented a pre-processing step and added an outlier detection scheme. That is, we implemented an LOF (Local Outlier Factor) algorithm [40] that measures the local deviation of a given data point with respect to its neighbours. Hereafter, we systematically use that filter to identify and filter out highly contaminated samples. The filter parameters (i.e., neighbourhood size, parameter for the Minkowski metric) were optimized by a cross-validated grid-search and evaluated using the silhouette score [41]. This pre-processing step is illustrated in Section 3.1. 

In a second step, we generated a new dataset for each pollen sample by binning randomly picked-up size distributions into cumulative ones. Overall, 30 cumulative size distributions were calculated for each pollen species. We implemented this oversampling [42] procedure to handle the problem of imbalanced data and to ensure there were enough counts in each size bin. Indeed, at least 20 particles per size bin are necessary to achieve the mean scattering properties of a given species [43]. Following that we concatenated the obtained datasets to form a unique dataset of 360 rows (30 cumulated size distributions × 12 pollen species) and 19 columns (19 size bins), on which we applied a row-normalization. Finally, we verified the clustering tendency of the resulting dataset by using the Hopkins statistic [44,45], and subsequently performed an agglomerative hierarchical cluster analysis using dedicated R packages [46,47,48,49]. We used this clustering approach as it presents the advantage of being less sensitive to outliers, relatively easy to implement, and offers the possibility to test multiple distance metrics before selecting the one that is most appropriate for measuring size similarities between our pollen samples. 

#### 2.4.2. Pollen Classification

So far, we have addressed the data from the channel one only and considered the grouping of the pollen samples based on the size information, which could represent a first level of pollen discrimination. Our second objective now is to investigate whether pollen species belonging to the same size cluster could potentially be distinguished from each other and from other types of aerosols by using the information contained in all 4 channels.

**Figure 3:**
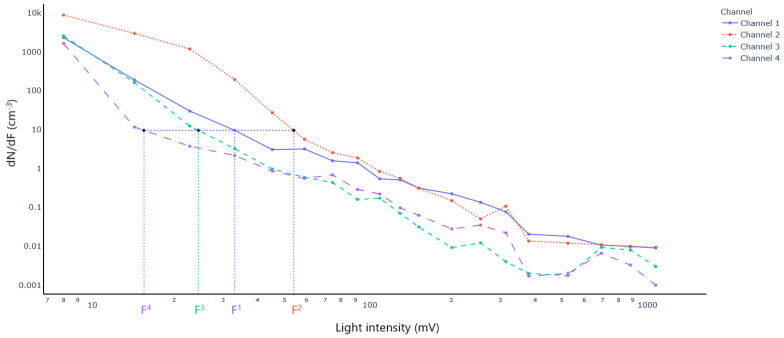
Principle for calculating the speciation indices using the light intensity distributions of the four channels (See Equations (1) and (2)).

This task can in principle, be achieved by feeding the raw data into a classifier and evaluating its learning performance in identifying each specie. However, the raw data (i.e., light intensity distributions), as provided by the 4 channels are count data with excess of zeros and highly skewed distributions. This in addition occurs in a high dimensional space, which makes the classification task challenging. A data transformation step was thus required before training any classification algorithm. We used a domain-expertise technique to reduce the initial space by computing what we refer to as the speciation indices. These speciation indices are at the core of our discrimination method and are calculated following the procedure described in [39]. An illustration of this procedure is presented in Figure 3, in which light intensity distributions of the four channels are plotted. 

The principle of the speciation index consists in determining for particles that have scattered light with an intensity of a known value $F^{1}$ at channel 1, the intensities *F*^2^, *F*^3^ and *F*^4^ of channels 2 to 4 that would have given the same number concentration as the one recorded by channel 1. Mathematically, we look for $F^{c}$ as follows:

(1) $${\left(\frac{dN}{dF}\right)}_{1}\left(F^{1}\right)={\left(\frac{dN}{dF}\right)}_{c}\left(F^{c}\right),\;c=2,3,4$$

where c stands for channel, $F^{1}$ is a known value corresponding to the lower bound of the light intensity bin in which the particles to characterize were counted, and ${\left(\frac{dN}{dF}\right)}_{1},{\left(\frac{dN}{dF}\right)}_{c}$ are the light intensity distributions of channels 1 to 4 respectively. 

A speciation index $S^{c}$ is then defined as the ratio:

(2) $$S^{c}=\frac{F^{1}}{F^{c}},\;c=2,3,4$$

The pairwise ratios of the speciation indices are also computed before obtaining what we shall refer to as the optical signatures. An optical signature is thus a vector of 6 elements expressed as:

$$\left(S^{2},S^{3},S^{4},\frac{S^{2}}{S^{3}},\frac{S^{2}}{S^{4}},\frac{S^{3}}{S^{4}}\right)$$

where $S^{2},S^{3},and\;S^{4}$ are respectively the speciation indices obtained thanks to channels 2, 3, and 4.

We generated large datasets containing the optical signatures of species belonging to the same clusters/size classes using an oversampling procedure. In addition, we used the optical signatures of droplets, as well as carbonaceous and mineral particles for the classification task.

After this data transformation step, we wanted to model the probability of each particle belonging to a given pollen species or aerosol type. For this purpose, we calibrated a logistic regression model to predict a given pollen species by using the optical signatures as input features. We implemented this algorithm using a One versus One strategy as proposed by [50]. The decision threshold (i.e., probability threshold) was optimized for each binary classifier with the objective of maximizing the F1-score metric and a repeated 10-fold cross validation was also systematically undertaken to assess the ability for each classifier to generalize to new unseen data. We evaluated the performances of our classification algorithm by using the recall metric. Finally, we aggregated the results, and presented them in confusion matrices for each size cluster.

## 3. Results

### 3.1. Particle Sizing

The first part of this study entails the evaluation of the size information that is provided by Beenose. As outlined in the previous section on methods, a pre-processing step is necessary to reduce the contamination caused by ambient particles, debris, and aggregates.

**Figure 4:**
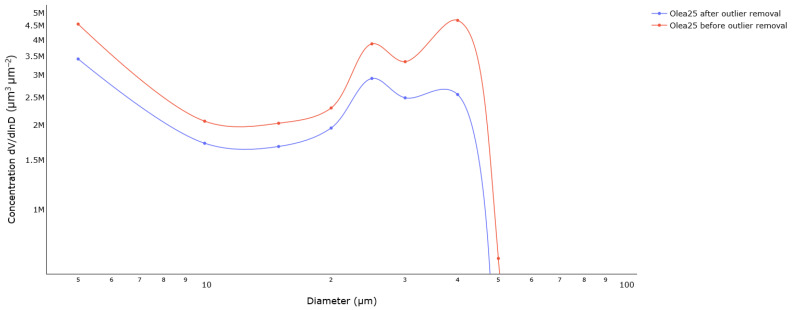
Volume size distributions of *Olea* measurement session before and after the outlier treatment procedure.

The effect of this procedure is illustrated in Figure 4 for *Olea* pollen grains. The LOF algorithm acts as expected by catching the outliers, which is confirmed by the volume size distributions before and after outlier removal. In particular, Beenose detected two modes in the initial volume size distribution, which are located at 25 µm and 40 µm. The first mode matches the theoretical diameter of *Olea* pollen grains while the second one is possibly due to aggregates. Notice how the second peak is less pronounced after outlier removal. This behaviour was observed with all the pollen samples, except for *Anthoxanthum*, *Fraxinus*, and *Quercus*, as shown by the average silhouette scores (Table 2), which are lower than 0.5.

Now that we have removed the outlier data, we proceed by determining whether the count data can be clustered with respect to the size of pollen grains and seek the optimal number of clusters that are needed to describe these data efficiently. The Hopkins statistic computed on the entire dataset gives a value of 0.99, which suggests that the data can be clustered. A voting scheme over more than 25 statistical indices computed on a varying number of clusters reveals that the optimal number of clusters is three.

**Figure 5:**
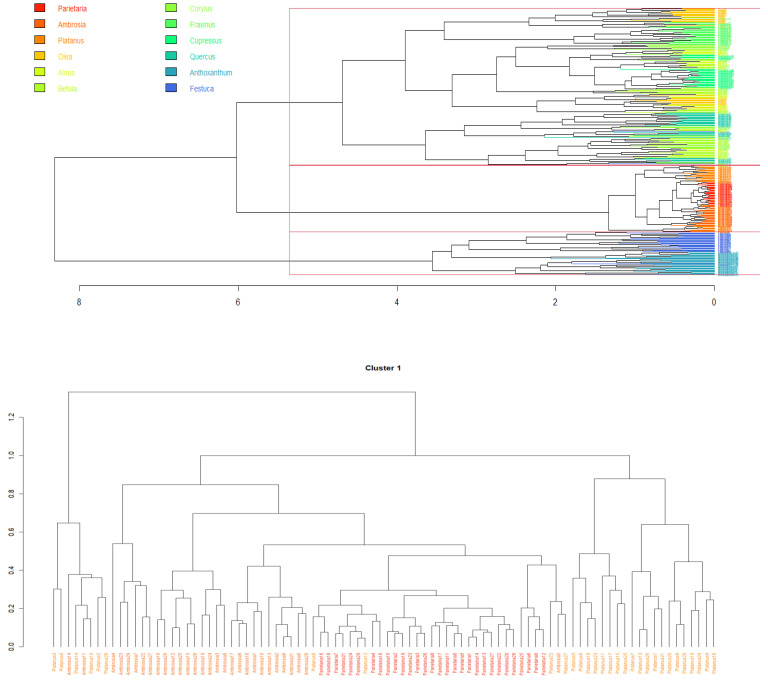

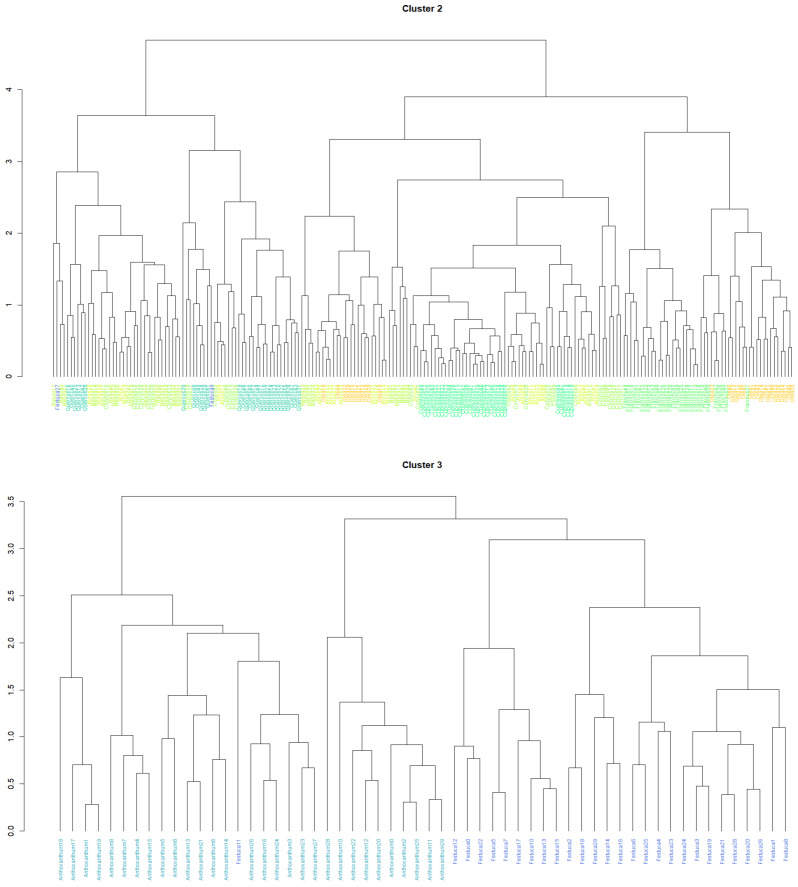
Dendrogram of the size based hierarchical clustering. The first panel shows the overall dendrogram with the 3 obtained clusters. The 3 other panels represent excerpts and zoom into each of the 3 clusters. Note that pollen measurements were coloured from red to blue in the ascending order of diameter, as provided by the supplier.

**Figure 6:**
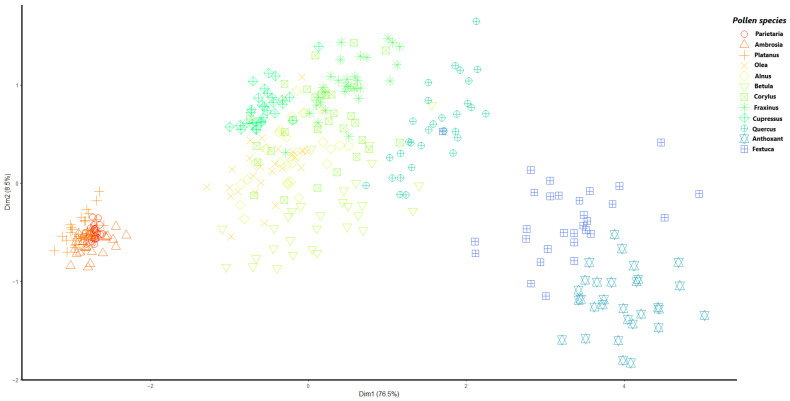
Dimensionality reduction of the pollen size information as provided by Beenose: the optical signatures are projected on their two principal axes, as obtained by PCA. Three groups can be distinguished: small size pollens (left), medium size pollens (middle), and pollens with the largest diameters (right).

To assess Beenose’s ability to provide relevant size information, we use hierarchical clustering analysis with Euclidean distances and a complete linkage. In Figure 5, we present the results of this analysis by means of a dendrogram. For easier visualization, a principal component analysis (PCA) of the observations is displayed in Figure 6, using a specific symbol for each pollen species label with the same color scale as in Figure 5. 

Interestingly, both figures show that the pollen species which are considered in this study can be grouped according to their theoretical diameters. The first cluster consists of small sized pollen species and contains: *Parietaria* (13 to 15 µm), *Ambrosia* (18 to 21 µm) and *Platanus* (22 µm). The second cluster comprises most of the species, which are the medium-sized ones: *Olea* (25 µm), *Alnus*, *Betula*, *Corylus*, *Fraxinus*, *Cupressus* (27 to 30 µm) and *Quercus* (36 µm). Finally, the pollen species with the largest diameters, namely *Anthoxanthum* (37 to 41 µm) and *Festuca* (42 to 48 µm) are grouped into cluster 3. 

### 3.2. Pollen Classification

**Figure 7:**
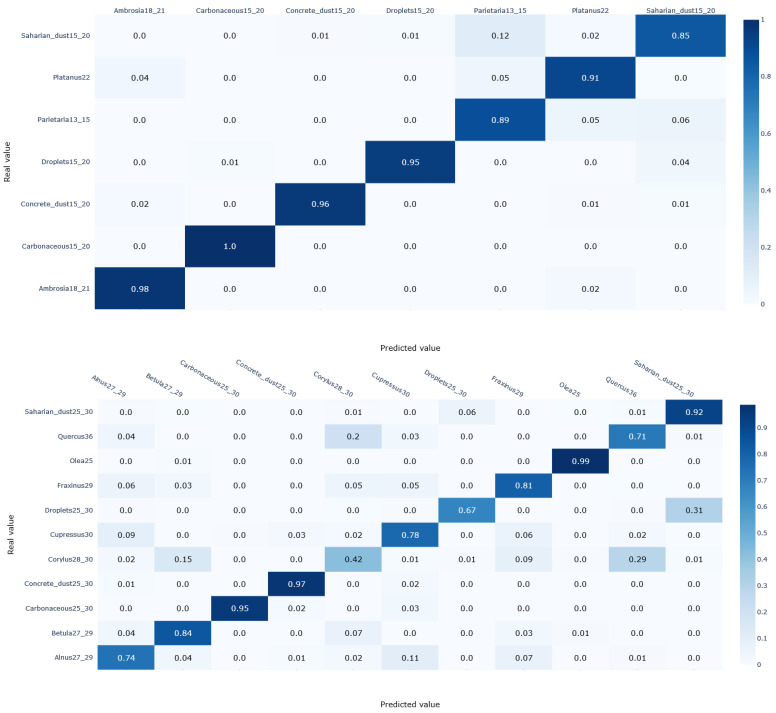

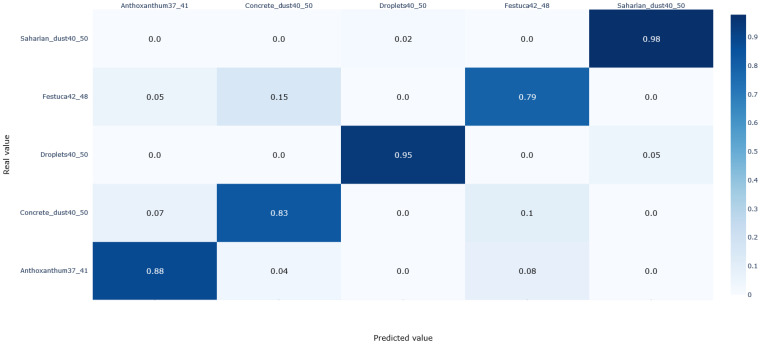
Confusion matrices of the predictions obtained by the logistic regression trained on the optical signatures of species that belong to the same size cluster.

The core part of our study is now the identification of pollen species based on their optical properties. First, we consider a preliminary step in which we perform a quick comparison of different classification algorithms of the python scikit-learn library (Logistic regression, Support vector machine, Multi-Layer Perceptron, and K-nearest neighbours) using default settings. Although Support vector machine shows slightly better accuracy (2% better), we opt for a logistic regression model as it has the advantage of being easier to interpret. This model was thus trained and validated following the procedure described in the Methods Section. The classification results are displayed in Figure 7 in terms of confusion matrices for each size cluster. 

The confusion matrices in Figure 6 show that that droplets, carbonaceous and mineral particles possess specific optical signatures with prediction scores (i.e., recall) exceeding 95% regardless of the size cluster they belong to. The only exception occurs with Saharan dust particles in the first cluster and concrete dust particles in the third cluster, with acceptable scores of 85% and 83%, respectively. Note that the optical signatures of carbonaceous particles are not included in the training routine of cluster 3 since they are rarely present with similar sizes in ambient air. In addition, most of the pollen species covered in this study are predicted with a recall above 85%, especially in the first and third clusters. The remaining species are correctly identified with a rate varying from 70% to 85%, except for *Corylus*, for which the recall is only 42%.

## 4. Discussion

The size-based clustering approach resulted in a grouping of the pollen species into three meaningful clusters, in agreement with the theoretical expected sizes, except for *Quercus*. The supplier specifies a diameter of 36 µm for this species, which is close to that of *Anthoxanthum* (37 to 41 µm). Surprisingly, *Quercus* was assigned to cluster 2, where the theoretical diameters range from 25 to 30 µm, instead of cluster 3, where the theoretical diameters range from 37 to 48 µm. One possible explanation for this result is the non-spherical size of the pollen grains, whose size along the polar and equatorial axes has been reported to be between 26 and 35 µm for *Quercus* [51]. Meanwhile, the clustering result confirms the hypothesis of retrieving size information at small scattering angles [38], namely channel 1 for Beenose.

When it comes to recognizing the pollen grains and discriminating them from other aerosols, we conclude that our pretrained logistic regression model, which relies on optical signatures, performs well globally. The data collected by the sensor has made it possible to discriminate between pollens on the one hand, and droplets and carbonaceous particles on the other hand. Indeed, the almost perfect prediction scores of these non-pollen particles show that their optical signature is very distinct from pollen optical patterns. This is not surprising since water droplets are optically transparent, and carbonaceous particles optically strongly absorbing [39]. However, confusion may occur with mineral particles that are sometimes misclassified as pollen species, which suggests that some pollen grains are semi-transparent.

Regarding the discriminative power of the instrument between different pollen species, the scores obtained were very promising, with 5 species out of 12 exceeding a prediction score of 85%. The algorithm performed slightly worse for the rest of the species, but the scores were still satisfactory, ranging from 70% to 85%. However, some pollen species had a similar optical signature and were therefore misclassified. Such misclassifications mainly affected *Corylus*, which was correctly detected in only 42% of the cases—29% were mistakenly labelled as *Quercus* and 15% as Betula. Likewise, 20% of *Quercus* were misclassified as *Corylus*. Such confusions are in line with those reported by [35] who identified the species of the Betulaceae family (*Alnus*, *Betula*, and *Corylus*) and *Quercus* as belonging to the same group, making it difficult to distinguish them. However, unlike with Beenose, these results were obtained with fluorescent spectra and therefore are not strictly comparable. 

Note there is also a difference of prediction scores between clusters 1 and 3, and cluster 2. The latter contains more species in comparison with the two others, which highlights the dependence of the quality of identification on the number of species to monitor, especially in the medium-sized cases. As the focus of our study is on the most allergenic ones, more confusions would probably have arisen if the focus had been on a larger number of species, for example by considering additional species with a low to moderate allergenic potency. Such confusion can be eliminated or at least reduced in a real-time operational context by having recourse to additional parameters such as the historical seasonality patterns or plant cover. Importantly, several confusions are not critical from a health point of view, since they involve taxa that have similar allergenic profiles (i.e., *Corylus* and *Betula*).

Finally, although the pollen samples used in our study were all dried by the supplier, they still varied in size, shape, and surface properties, offering the possibility to test the ability of the instrument in particle sizing and separating the samples based on their optical properties, and also to build a calibration dataset. Such a calibration dataset should be extended at a later stage with freshly released/hydrated pollen grains to anticipate pollen identification outdoors under varying weather conditions leading to different pollen states.

## 5. Conclusions

In this paper, we have investigated the ability of the Beenose sensor to deliver relevant information in terms of pollen identification based on laboratory measurements. Our results show that the sensor is able to deliver the following:

1- A consistent clustering of the pollen species based on their size properties.
2- An accurate separation between pollen and non-pollen particles.
3- A correct recognition of the pollen species with 9 out of 12 of the species covered in our study having a prediction score above 78%.

Our analysis also reveals some confusions either between some pollen species and mineral particles, or between pollen species that have a similar optical signature. This highlights the need for using data cleaning processes to reduce the effect of mineral pollution. Additional input parameters (pollen calendars, plant cover inventories, satellite imagery, etc.) are required to further constrain the classification for operational purposes. To this end, several sensors have already been deployed at different locations in France and in Belgium, and further developments of the instrument and the classification algorithms are in progress to improve the quality of the monitoring. The results of this validation campaign including inter-comparison with Hirst-type traps will be presented in upcoming papers.

## Figures and Tables

**Table 1:** List of the pollen species used in the study; the diameters provided by the supplier were determined under microscope at 400 and/or 1000 magnification in the form of either a range or a mean.

Common Name	Latin Name	Theoretical Diameter	Number of Samples	Total Number of Grains
Alder	*Alnus glutinosa*	27 to 29 µm	216	2593
Sweet vernal grass	*Anthoxanthum odoratum*	37 to 41 µm	171	6988
Ragweed	*Ambrosia* *artemisiifolia*	18 to 21 µm	179	1350
Birch	*Betula pendula*	27 to 29 µm	209	1789
Hazel	*Corylus avellana*	28 to 30 µm	242	1403
Cypress	*Cupressus sempervirens*	30 µm	189	3896
Fescue	*Festuca pratensis*	42 to 48 µm	195	5196
Ash	*Fraxinus excelsior*	29 µm	198	2462
Olive tree	*Olea euopaea*	25 µm	231	1244
Wall pellitory	*Parietaria officinalis*	13 to 15 µm	299	5150
Plane tree	*Platanus acerifolia*	22 µm	201	634
Common oak	*Quercus robur*	36 µm	203	3557

**Table 2:** Average silhouette scores after partitioning the measurements of each pollen sample into inliers and outliers.

Pollen Species	Silhouette Score
*Alnus glutinosa*	0.6
*Anthoxanthum odoratum*	0.41
*Ambrosia* *artemisiifolia*	0.63
*Betula pendula*	0.54
*Corylus avellana*	0.59
*Cupressus sempervirens*	0.54
*Festuca pratensis*	0.65
*Fraxinus excelsior*	0.28
*Olea euopaea*	0.6
*Parietaria officinalis*	0.59
*Platanus acerifolia*	0.5
*Quercus robur*	0.47

## Data Availability

The light scattering curves obtained with the PROGRA2 instrument used for the Beenose conception are available at: https://www.icare.univ-lille.fr/progra2-en (accessed on 6 September 2022) All other data and algorithms used in this study will be available at the same repository following a 24-month embargo from the date of publication to allow for commercialization of research findings.
